# Pathological fixation on shared beliefs: a review and bibliometric analysis of extreme, overvalued and delusion-like beliefs

**DOI:** 10.3389/fpsyt.2025.1715886

**Published:** 2025-12-04

**Authors:** Kolbrun Kristinsdottir, Mark Freestone, Alan Underwood, Julia Ebner

**Affiliations:** 1 Centre for Psychiatry and Mental Health, Wolfson Institute of Population Health, Faculty of Medicine and Dentistry, Queen Mary University of London, London, United Kingdom; 2 The Education and Training Directorate, Tavistock and Portman NHS Foundation Trust, London, United Kingdom; 3 Stalking Threat Assessment Centre (STAC), North London NHS Foundation Trust, London, United Kingdom; 4 Calleva Centre of Evolution and Human Sciences, University of Oxford, Oxford, United Kingdom

**Keywords:** overvalued beliefs, extreme beliefs, delusion-like beliefs, fixation, violent extremism, lone-actor violence, forensic psychology

## Abstract

**Introduction:**

As researchers, practitioners, and policymakers highlight the changing landscape of security concerns, the growing domestic terrorist threat of radicalised lone actors cannot be overlooked. Extreme, overvalued and delusion-like beliefs are one piece of the puzzle, as adherence to shared extreme beliefs and conspiracy theories has become increasingly concerning in recent years. While interest and awareness have grown, there is a notable lack of consistency in conceptualisation and terminology across disciplines. A bibliometric analysis helped highlight existing focus areas across disciplines, current gaps and opportunities for future research.

**Methodology:**

The Web of Science Core Collection was searched to identify publications on extreme overvalued beliefs, overvalued ideas, delusion-like beliefs, extreme beliefs, extremist beliefs, radical beliefs and strongly held ideas from January 2005 to September 2025. Trends in publications, dates and contributing countries were explored, and all results were uploaded to VOSviewer. A map based on bibliographic data focused on a keyword co-occurrence analysis was then generated and analysed separately for each concept in the search, and then combined to explore research focus in medicine and social sciences.

**Results:**

The majority of publications were from the United States, followed by England, Australia and Canada. There was a gradual increase in publications from 2005, a spike in 2015 and a notably increased focus since 2020. Co-occurring keywords with the strongest links were different for the concepts searched, but overlapped significantly between overvalued and delusion-like beliefs and on extreme beliefs and radical beliefs. The focus was also different in psychiatry, psychology, neuroscience and medicine compared to social science disciplines (law, sociology, philosophy, anthropology and criminology).

**Discussion:**

This bibliometric review presents current knowledge, limitations, gaps, and recommendations for future studies on extreme, overvalued, and delusion-like beliefs. The review highlighted significant blurring of concepts between extreme, overvalued, and delusion-like beliefs and strongly held ideas that were striated along disciplinary lines. A need for greater consensus on the definitions and overlap of these terms was identified and discussed. By clarifying conceptual ambiguities, this review provides a foundation for developing cross-disciplinary consensus in threat assessment.

## 1 Introduction

“We see acts of extreme violence perpetrated by loners, misfits, and young men in their bedrooms, accessing all manner of material online, desperate for notoriety” (1).

These words from the Prime Minister of the United Kingdom, Keir Starmer, came following an attack in Southport, England, where an 18-year-old man stabbed 11 children and two adults during a Taylor Swift dance class, which resulted in the death of three young girls (2, 3). In his speech, Mr Starmer warned of the growing risk of radicalised lone offenders, who seem to be “fixated on violence for its own sake” (1). The Southport attack was meticulously planned by a young man who had a history of violence and alarming online behaviour, and had been referred to government-led P/CVE programmes three times for worrying and violent behaviour (2–4). Comparable attacks perpetrated by fixated lone offenders have been observed internationally, including in the United States (5–8), the United Kingdom (2, 9), Denmark (10), Norway (11, 12), New Zealand (13) and Canada (14). The details and modus operandi differ across cases but range from school shootings and lone-wolf terrorism, frequently referred to as ‘lone-actor grievance-fuelled violence’ (15–17). The ‘umbrella term’ of lone-actor grievance-fuelled violence was introduced based on recent and emerging research findings highlighting the significant commonalities of these offenders (18, 19). Cases of lone-actor grievance-fuelled violence have been increasingly connected to shared ideological subgroups such as QAnon, Neo-Nazism, Proud Boys, jihadism, anti-LGBTQA+, and the Involuntary Celibates (Incels) (20–23).

This development marks a change in emerging terrorist activity, with a shifting focus on threats posed by lone actors and a growing concern about individuals with extreme, overvalued and delusion-like beliefs (4). This observation has been highlighted by the European Union (24), the Federal Bureau of Investigations (25, 26), Europol (27), and covered in the 53^rd^ (28, 29), 54^th^ (30, 31), and 55^th^ (32) annual meetings of the American Academy of Psychiatry and the Law. Not only is the focus shifting to lone actors, but the previous role of structured ideologies, often based on traditional religious or political agendas, is transforming with the internet to what is frequently referred to as “hybrid ideologies” (33) or “salad bar extremism” (34, 35). There is an increase in incoherent motivations that are difficult to conceptualise and unravel as they consist of a complex combination of ambiguous and fused beliefs, exacerbated by grievances and personal vulnerabilities, which directly affect behaviour and identity (34). As the Prime Minister noted, lone offenders often get inspiration from terrorist groups based on similar beliefs and ideology, but their primary fixation appears to be on violence (1). This was emphasised following the Southport attack, outlining the need for improved risk assessments of radicalised individuals with no coherent ideology (3).

### 1.1 Pathological fixation

Fixation is a significant risk factor often driving ideological or grievance-fuelled commitment, observed in many mass casualty attacks, including lone-actor terrorism, school shootings, workplace shootings and other targeted attacks (15, 17, 19, 36–38). It refers to an increasingly pathological preoccupation pursued to an abnormal and intense degree, often leading to social withdrawal and deterioration of normal functioning (36, 39). The fixation can revolve around a person, event, idea, or belief (36, 39), and in numerous cases, fixation can focus on violence and gore (26). The content of the fixation may not be the central factor of concern, as various religious or political ideologies may influence this development, tap into delusions, or exist along with a complex interplay of grievances and mental illness (40). Understanding and recognising the level of risk a fixated individual poses is determined by their motivation and symptomology (41).

Fixation, on its own, does not predict violence, as normal fixation can be part of everyday life. Many develop a fixation with their spouse, family, children, or even a sports team or a celebrity, which never develops into pathological preoccupation (39). However, in more severe cases, especially when a belief co-exists with other factors that exacerbate the risk of violence (42), it can lead to serious attacks (15, 19, 36, 39, 43, 44). Meloy and colleagues (2011) presented the key characteristics of fixation as a warning behaviour of violence: *growing perseveration, increasingly strident opinion with an angry emotional undertone and negative characterisation of the object of fixation that has an impact on the family or other associates of the object of fixation and is typically accompanied by social or occupational deterioration* (45). This fixation frequently stems from an underlying mental illness, such as a psychotic disorder or delusional disorder (39), but it can also appear somewhere on a spectrum of pathological beliefs (46). Importantly, fixation can stem from beliefs shared with a subcommunity and can develop into something more overbearing, absolute and emotional over time, making the aetiology, underlying motivation, and development completely different (36). The distinction can be tricky, as the content and subsequent behaviour can, at surface level, seem similar.

### 1.2 Delusions, obsessions and overvalued ideas

Carl Wernicke originally introduced overvalued ideas to psychiatry in the late nineteenth century as an “*Ueber fixe Idéen*”, a shared pathological belief that aligned with an individual’s personality, preexisting values and experiences (47–51). Wernicke introduced overvalued ideas in the context of criminality, not as a symptom of a serious mental disorder, and argued that they should not be confused with insanity as they are neither delusional nor obsessional despite unorthodox content (47, 48, 50). They were believed to grow increasingly overbearing with time, becoming a major disruptor in the individual’s life, viewing great sacrifice as worthy of the cause, whether it be status, relationships, or assets (52, 53). Overvalued ideas are often associated with an abnormal personality (54), and the possessor was frequently referred to as a ‘fanatic’ in earlier writings and common speech (52). This is unsurprising as the overvalued idea was held with a passion that preoccupied the mind of the possessor to the extent that it could dominate their lives and influence behaviour to a pathological degree (48, 50, 52).

The content, presentation, and subsequent behaviour associated with a delusion or obsession can be similar to one instigated by an individual with shared overvalued beliefs, but the cognitive components, psychopathology, and aetiology are distinct (36, 51). Delusions are false and unshared beliefs that remain fixed despite clear evidence of the contrary (36, 47, 50). They are idiosyncratic and reflect some incorrect inference about a shared reality held with intense conviction, contradicting their education and cultural upbringing and what other members of their culture or subculture view as correct (49, 52, 55). They are also frequently accompanied by other markers of psychosis, such as auditory or visual hallucinations (56). An individual with overvalued ideas can have a somewhat coherent explanation for their belief in line with their value and personality, whereas the content and rationalisation of delusions tend to be less plausible and reasonable (56). Furthermore, the onset of a delusion is usually sudden, not gradual, as is observed with shared overvalued beliefs (36, 56).

The third cognitive-affective driver of fixation is obsessions, which are also frequently confused and used interchangeably with overvalued ideas (57). Carl Wernicke was the first to distinguish between obsessions and overvalued ideas in psychiatry and stated that overvalued ideas, to some extent, made sense and were in line with the individual’s personality (48, 49). Obsessions, however, are defined as persistent and intrusive ego-dystonic thoughts, images and/or urges which cause significant distress to the individual. They are experienced as unwanted, frequently anxiety- and shame-provoking (55, 58). The thoughts are suppressed and resented, causing the individual significant distress, which leads many to perform mental or behavioural compulsions to neutralise their experiences, sometimes developing into Obsessive Compulsive Disorder (36, 58). Overvalued beliefs are ego-syntonic and in line with the individual’s values, usually rejoiced, amplified and protected by the possessor (36, 53), while obsessions contradict the individual’s beliefs and values, leading to negative emotions and shame (36, 50, 59).

An accepted notion of overvalued ideas is that preoccupation is held to a less-than-delusional intensity (54, 58, 59). Interestingly, however, while delusions are believed to be held to a higher strength, overvalued ideas can more severely preoccupy the person’s mental life (54). Individuals with delusions are more likely to be unconcerned with other people’s opinions, while those with overvalued beliefs tend to care considerably about other opinions and are more likely to be displeased (or pleased) with disagreement (56). Overvalued beliefs, in contrast to delusions and obsessions, have long been misunderstood and widely under-researched as they have almost exclusively been studied in individuals with eating disorders (48, 50, 54). That being said, overvalued ideas in anorexia nervosa have substantial differences from delusions and shared similarities with overvalued beliefs proposed by Wernicke and later Rahman and colleagues. Specifically, overvalued ideas are deeply emotional, with a significant impact on thoughts and behaviour, and develop over time (60). While overvalued ideas are emotional, dominating, non-objective, and one-sided, they are not entirely irrational, as is often the case with delusional thinking (61).

McKenna described the phenomenology of overvalued ideas as “(*ideas that) seem to combine the unlikely elements of non-delusional conviction, non-obsessional preoccupation and non-phobic fear*” (47, p. 583). A pathological belief is, *generally*, not considered delusional if shared with others (52, 62, 63). While this distinction worked reasonably well before technological advances, the complexity of a virtually connected world severely blurred this assumption, with online subcommunities consisting of relatively large groups sharing eccentric ideas and beliefs (52). Thus, there are several complexities introduced by modern technology, including the ambiguity it presents for our information processing and decision-making capabilities, and the accompanying difficulties in proper psychiatric diagnoses and treatment, especially in cases of non-bizarre delusions (58). It can be challenging to distinguish drivers of fixation solely based on content or behaviour, but the underlying mechanisms, cognitive elements, and risk factors differ substantially, which is important to recognise for prevention strategies and subsequent treatment (36). Preliminary evidence suggests that forensic psychiatrists have a substantial ability to distinguish accurately between fixation driven by delusions, obsessions and overvalued beliefs when given a proper definition and clinical guidelines (64).

### 1.3 Extreme overvalued beliefs

The discrepancies in definitions of overvalued beliefs remain a complex factor in psychiatry and psychology (54). The aforementioned conceptualisation differs significantly from the current definition offered in the Diagnostic and Statistical Manual of Mental Disorders 5^th^ Ed. (DSM-5), more widely accepted in American psychiatry (50, 54, 64). According to the DSM-5, overvalued ideas are abnormal and irrational beliefs that remain persistent despite *not* being accepted within a person’s culture or subculture (52, p. 826). Thereby, the sharing of the overvalued idea was disregarded in later psychiatric writings and abandoned entirely in American diagnostic manuals, which focused mainly on the abnormality of the belief (50, 54, 58). The definitions offered in the DSM-5 are heavily debated and criticised by practitioners and within the wider scientific community due to the failure to meet the changes associated with a connected world. Especially considering the growing social threat of domestic terrorism posed by violent subcommunities that prey on the vulnerability of radical lone actors (64–66) as the shared aspect combined with the intensity was hypothesised to increase the likelihood of a destructive outcome (52). The aforementioned definition of non-shared overvalued ideas in the DSM-5 fails to capture this critical aspect.

An alternative use for overvalued ideas was presented by the World Health Organisation (International Classification of Diseases 11^th^ revision; ICD-11), where an overvalued idea could be recognised, even if it was built on somewhat conventual content (such as religious ideas or political opinions) if it was held with such intensity that it disrupted and severely occupied the person’s life (53, p. 696). While the ICD-11 definition is vague, it coincides more closely with the definitions offered in many notable European psychiatric writings (54), including PJ McKenna (48), Andrew Sims (51, 67), Karl Jaspers (47), Oliver Freudenreich (52), and in earlier publications of the Oxford Textbook of Psychiatry (68). Wernicke’s conceptualisation was reintroduced to the study on threat assessments by McHugh following the 9/11 terrorist attacks (53) and later adapted by Rahman and colleagues (64–66) with the definition of an extreme overvalued belief (36, 66).

Drawing on decades of research in psychiatry and psychology and the aforementioned conceptualisations in European psychiatry (48, 50, 51, 53, 54, 66), *extreme overvalued beliefs* were introduced as one of three cognitive-affective drivers of fixation. They were introduced alongside delusions and obsessions, to capture and partly explain the growing risk of overvalued ideologies shared within subcultures in modern society (21, 65, 69). Rahman, Meloy and colleagues introduced (2016, 2018, 2019, 2020, 2021) the term in hopes of improving clinical and forensic evaluations by providing an alternative psychiatric explanation for the unwavering and pathological yet non-delusional fixation seen in cases of targeted violence (70). By definition, extreme overvalued beliefs are shared beliefs or ideologies that are absolute, simplistic, and binary in nature, difficult to disprove and rigidly held despite countering evidence. They are emotionally heavy, celebrated and protected by the individual who has them and frequently fuelled by a personal grievance, growing more overbearing and intense over time (36, 50, 65, 66).

As highlighted for fixation, extreme overvalued beliefs would not always increase the risk of violence, but recent evidence suggests that when they are combined with perceived outgroup threat, ‘othering’ or demonisation of the outgroup, and endorsement of violence, in addition to the identity fused with the wider perceived in-group, the propensity for violence increases (42). Distinguishing drivers of fixation and the separation of overvalued ideas from delusions and obsessions remains an ongoing and debated topic, with experts often struggling to make the distinction (46, 71–73). However, for the intersection of psychiatry and law, this remains a critical and growing concern as individuals with delusions can be exempt from legal responsibility due to a serious mental illness severely limiting their cognitive capacity, which is not the case for individuals with shared overvalued beliefs (36, 74).

### 1.4 Delusion-like beliefs

Challenging the often-accepted categorical view of beliefs in psychiatry, delusion-like beliefs have been described within psychiatric texts as beliefs that do not meet the criteria of delusion but, at face value, could be prone to misdiagnosis (73). However, similar to overvalued beliefs, delusion-like beliefs do present conceptual and diagnostic complexity. In earlier psychiatric writing, Karl Jaspers described the existence of normal, overvalued, delusion-like and delusional beliefs. A delusion-like belief has also been referred to as a ‘secondary delusion’. It would not be completely contradictory to an individual’s education, culture and upbringing, as is often the case with delusions that were described as ‘ununderstandable’ (75, p. 97). Contrary to delusions, delusion-like beliefs and overvalued ideas share clear communities in alignment with background and insight, especially one’s personality. Distinguishing between delusion-like beliefs and overvalued beliefs would be a complex process and Karl Jaspers did not focus much effort on the distinction between the two as there was ‘little to be gained’, whereas understanding how both differ from a delusion remained a vital psychiatric question, as delusion was a symptom of disease (47, 75).

The current categorisation of delusion-like beliefs was intended to help explain a variety of beliefs that are at risk of “slipping through the cracks” in current diagnostic manuals (73). In particular, this may include beliefs that develop online and are shared with others, have no direct delusional component, but do not align with logical, rational, or evidence-based beliefs (72, 73). Similar to overvalued beliefs, delusion-like beliefs have been described in body dysmorphic disorders (76) and in relation to conspiracy beliefs and shared subcultural ideologies (21, 22, 65, 69). Currently, the most cited and common type of delusion-like beliefs are conspiracy theories, which often present a diagnostic complexity for psychiatrists and psychologists. The content can appear delusional, but they are usually recognised as extreme or overvalued beliefs which are shared with a subgroup, often traced back to misinformation (62, 69, 72, 73, 77–79) and strongly associated with extremism at different ends of the political spectrum (80). Conspiracy ideation has been rising following the COVID-19 pandemic and is influential in many extreme and radical subcultures today, including QAnon and Sovereign Citizens (21, 73, 81, 82).

### 1.5 Integrating perspectives

Extreme overvalued beliefs undeniably have clear commonalities with both delusion-like beliefs and radicalised extreme beliefs, as has been previously highlighted (63, 69, 72, 73). Extreme overvalued beliefs have been described as the most common cognitive-affective driver of fixation in targeted violence (70), and some experts believe that extreme overvalued beliefs are more prevalent than delusional disorder (83). Still, the terminology and conceptualisation has attracted criticism (63, 73, 84). It has been argued that the conceptualisation and definition is too broad, encompassing different types of beliefs and blurring important distinctions (78), in addition to providing little justification for the terminology of ‘extreme’, severely limiting its usefulness for violent extremism and criminal insanity (84). Similar issues apply to the utilisation and guidance in clinical practice (63), as identification of extreme overvalued beliefs has been argued to be limited to *post hoc* assessments of criminal behaviour (69). Distinguishing extreme, delusion-like or overvalued beliefs from delusions based on the DSM-5 criteria of “fixed, false and unshared” (58) can be ambiguous and difficult to navigate (63, 73). Thereby, it has been criticised that despite its relevance in modern threat assessment, the term extreme overvalued beliefs provides little guidance or validation in a clinical and forensic setting (63, 72, 73, 78).

Many terms have been introduced to improve our understanding of unorthodox, intense and overbearing beliefs, thoughts and ideas that do not fit the criteria of a delusion. Some core characteristics appear to stay consistent across different terminologies; beliefs that are shared with subcommunities, develop over time, have no direct delusional or obsessional component, and, to some extent, align with personality, experiences, values and grievances (36, 50, 52, 54, 65, 72, 73). The discrepancy and ambiguity in findings, definitions, and conceptualisation make it close to impossible to estimate the prevalence and violence risk associated with overvalued beliefs or explore factors that distinguish them from delusions and obsessions (50, 63, 66, 72, 73). The original conceptualisation of overvalued ideas and the later introduced extreme overvalued beliefs by Rahman, Meloy & colleagues aid in the distinction between delusional and shared beliefs, which has long been difficult to unravel (83). However, despite efforts, the concept remains debated (48, 72, 73, 82).

Extreme overvalued beliefs (50, 64), delusion-like beliefs (72, 73), conspiracy theories (82), extremist and radical beliefs (63, 85), and a wider application of overvalued ideas (86) greatly overlap across disciplines. With this in mind, given the existing debates and inconsistencies in terminology, the current paper presents a bibliometric review of existing research on extreme, overvalued, radical and delusion-like beliefs within medicine, psychology, neuroscience and in other social science disciplines, where these beliefs have been studied significantly but using distinct terminology. The analysis will present a map of keywords to outline co-occurring presentations across disciplines to highlight inconsistencies, limitations and points of focus for future research. Identifying such inconsistencies in the language and application of these concepts might be the first step to help advance cross-disciplinary approaches that can help improve our understanding of the drivers of rising levels of lone-actor violence. Ultimately, connecting the evidence across different disciplines is needed to develop more effective prevention and intervention strategies.

## 2 Method and materials

### 2.1 Design and data collection

The data was collected on the 10^th^ of September, 2025, using the Web of Science Core Collection, as done in previous studies with a similar focus (87–90). To ensure validity across databases, a partial cross-database comparison was conducted by two authors (KK and JE) on SCOPUS. The cross-referencing across SCOPUS and Web of Science was largely consistent and overlapped significantly, with the comparison metrics between 50% and 93%^1^. The decision not to include a multi-database search in the analysis was due to the discipline-specific focus of the review. While most databases include the option of analysing discipline-specific research, a detailed analysis of multiple databases would be prone to error as the subject areas can be categorised differently (e.g. specific subfields within medicine and social sciences). Web of Science is frequently utilised for bibliometric reviews as it provides an extensive database of interdisciplinary research. The database allows for a comprehensive review of current trends and data, while including the option of detailed discipline-specific analyses (88, 89) with a focus on top-tier journals (91).

The following keywords were entered into Web of Science and SCOPUS for the partial cross-source comparison: (extreme overvalued belie*) OR (overvalued idea*) OR (overvalued belie*) OR (extreme belie*) OR (extreme idea*) OR (extremist belie*) OR (extremist idea*) OR (delusion-like belie*) OR (radical belie*) OR (radical idea*) OR (strongly held idea*). Upon searching the database, the results were limited to research articles, reviews and book chapters in English, published since 2005. The focus of the review was to analyse recent publication trends to get an accurate representation of the relevance, including the effect of technological advances, and was therefore limited to the past 20 years. Research areas of interest were social and medical sciences, specifically psychology, psychiatry, neuroscience, medical ethics, legal medicine, law, philosophy, social science, international relations, sociology, criminology, behavioural science, social work, and anthropology. Trends in publications were analysed, with attention to dates, countries and keywords. The concepts were entered separately to explore the publications and co-occurring keywords within each field. Then, all terms were searched together but categorised into either psychology, psychiatry and medicine or law, sociology, anthropology, criminology, behavioural science and international relations.

### 2.2 Data extraction and analysis

VOSviewer was used to analyse and visualise the data (92). The software is designed to generate maps based on co-occurring factors, such as geography, journals, authors or keywords and is ideal for visualising bibliometric data (93). It allows researchers to create network maps that visualise data for a comprehensive exploration of a research field and focus on keyword co-occurrences. The software was suitable for the current review to examine keyword trends in research on extreme, overvalued and delusion-like beliefs, as well as explore emphasis across research disciplines where focus and terminology have been applied in different contexts (94). Thereby, following a search of relevant databases, all records were uploaded to VOSviewer, where a network visualisation map based on bibliographic data was generated, including a keyword analysis across publication trends to explore co-occurring keywords with the strongest data links.

To get a clear idea of the research focus, a bibliometric co-occurrence analysis was conducted in psychiatry, psychology and medicine and for other social science disciplines. Given the discrepancy in conceptualisation, this presented a better overview of current knowledge in these separate (albeit related) disciplines. All relevant files were uploaded simultaneously with a focus on the co-occurrence of all keywords. Before generating the map, a review was done of the keywords identified, and the thesaurus was fixed to generate a better representation of published research to provide a clear and representative overview of the findings. Keywords were manually removed using Microsoft Excel out of context (for example, “variable”, “correlation”, “review”) or clustered together based on synonymous meanings (for example, “conspiracy belief” and “conspiracy theory”^2^).

## 3 Results

### 3.1 Selection and retrieval of documents

**Figure 1:**
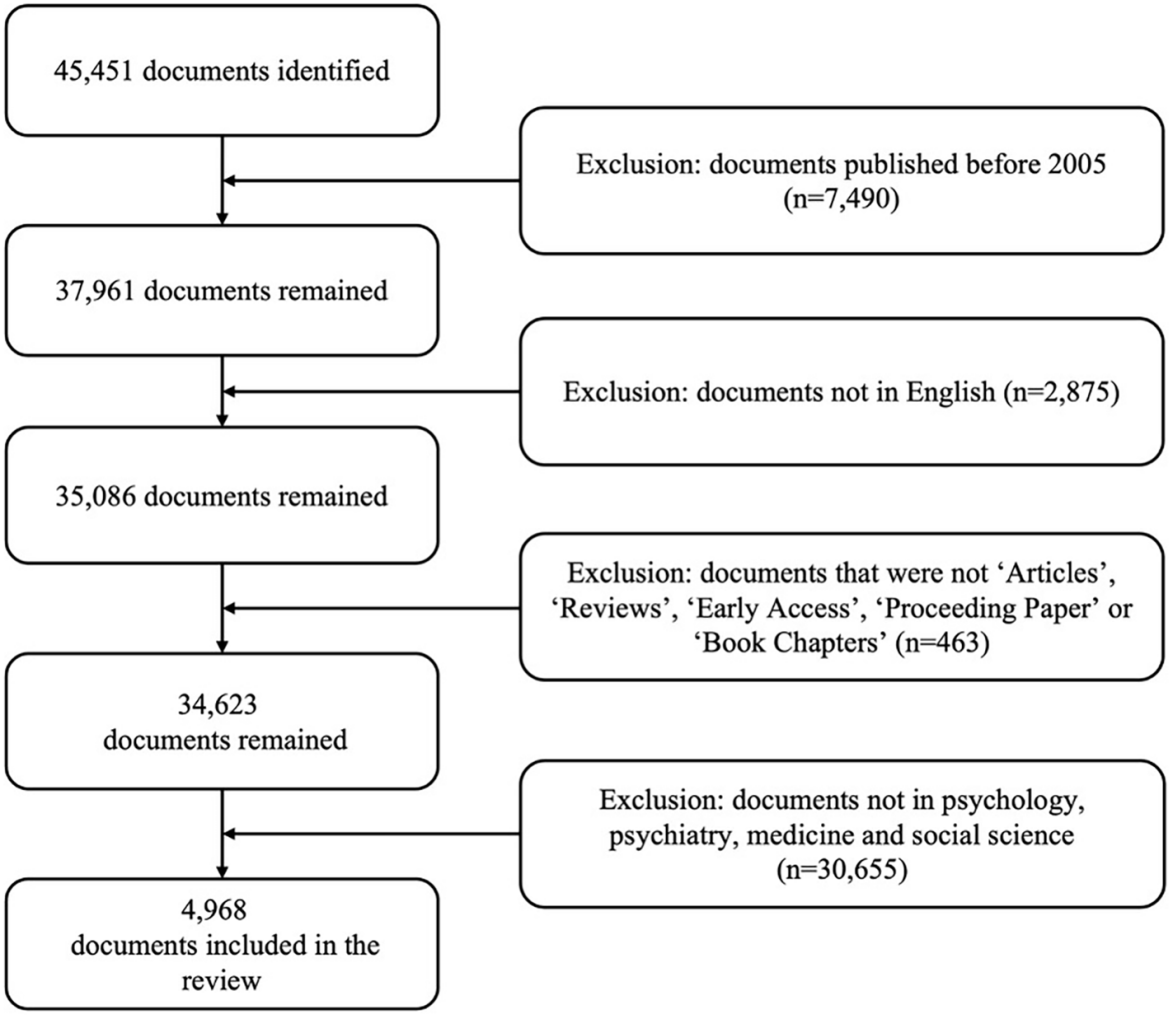
Flow diagram of papers included and excluded in the review process.

The search yielded a total of 45,451 results, but after a filtering process based on the aforementioned criteria, 4,968 documents remained (**Figure 1**).

### 3.2 Trends in publications

**Figure 2:**
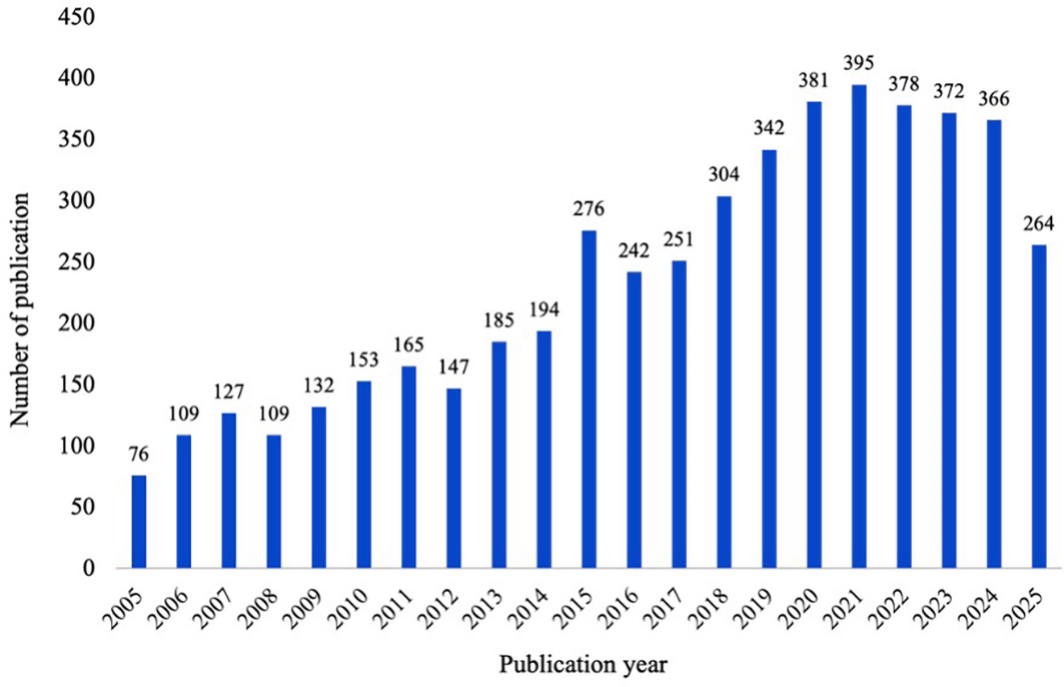
Trends in publication and coverage of extreme, overvalued and delusion-like beliefs since 2005.

The remaining papers were published from the 1^st^ of January 2005 to the 10^th^ of September 2025, with an increase in publication almost every year, with a spike in 2015 and a noticeable increase in the past five years. This indicated an increased focus on the topic in recent years (**Figure 2**)^3^.

**Table 1** displays the top 15 countries with the most publications on this topic since 2005^4^. The publications across these countries were 4,499 in total and represented 91% of all publications, with one-third coming from the United States.

**Table 1:** Publication across countries.

Countries	Number of publications	Percentage of publications
United States	1,604	33%
England	731	15%
Australia	309	6%
Canada	287	6%
Germany	247	5%
Netherlands	206	4%
Italy	192	4%
China	167	3%
Spain	151	3%
France	133	3%
Russia	123	3%
Sweden	102	2%
Belgium	85	2%
Scotland	83	2%
Israel	79	2%

**Figure 3:**
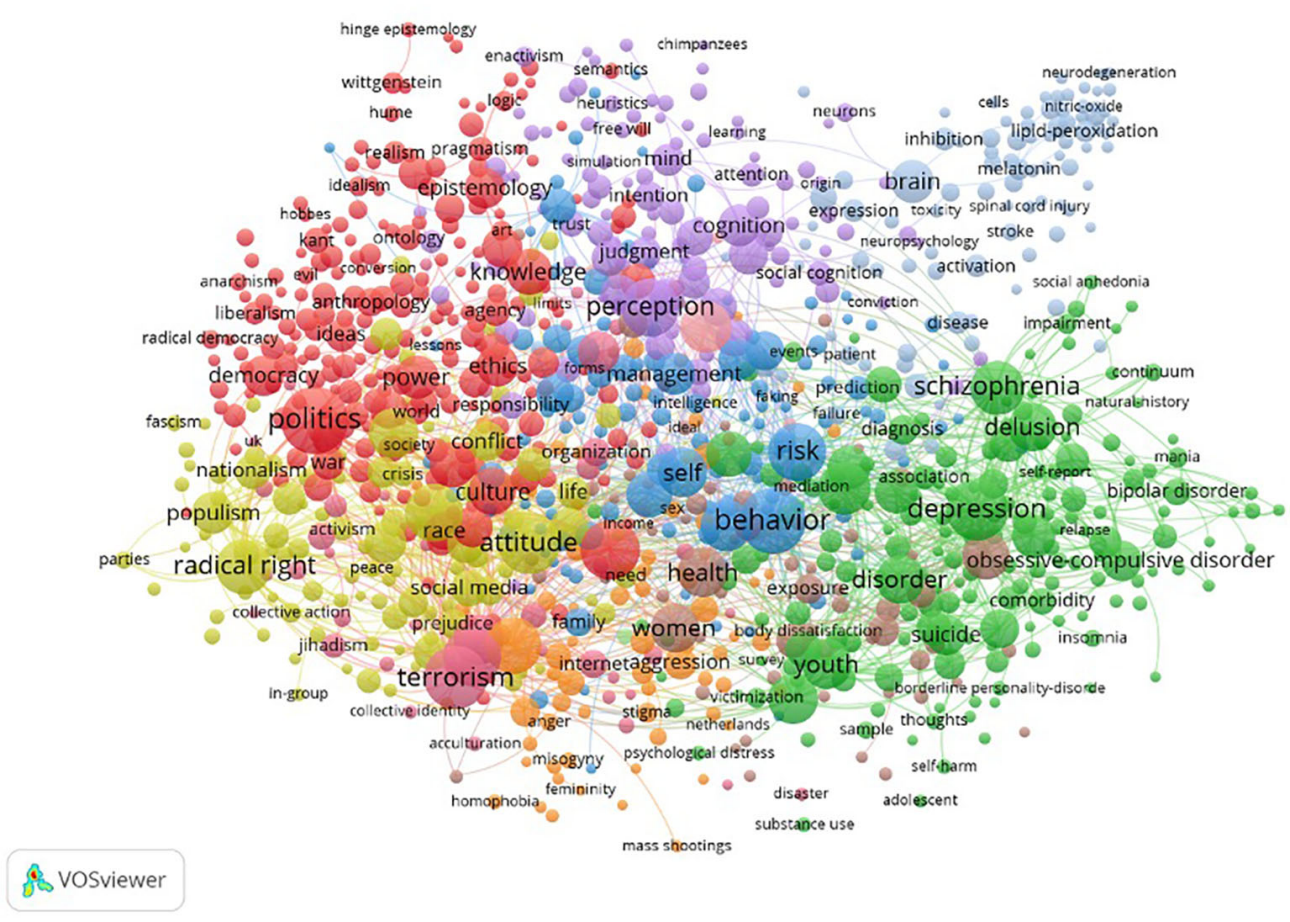
Keyword co-occurrence map on extreme, overvalued and delusion-like beliefs across all relevant scientific disciplines from 2005 to 2015.

*Note*. Mental disorders (green), violence and extremism (pink and orange), ideology and identity (red and yellow), cognition (purple and blue), medicine and disease (grey), overlapping keywords, a category of associated words that overlap significantly, including women, health and exposure (brown).

**Figure 3** presents the overview of the bibliometric co-occurrence analysis for all keywords listed above. In total, 19.009 keywords were identified, with 1,590 meeting the threshold of four or more mentions across studies. The co-occurring keywords with the strongest link were ‘Politics’, ‘Behaviour’, ‘Terrorism’, ‘Depression’, ‘Attitude’, ‘Gender’, ‘Radicalisation’, ‘Identity’, ‘Perception’, ‘Risk’, ‘Violence’, ‘Radical Right’, ‘Schizophrenia’, ‘Childhood and Youth’, ‘Personality’ ‘Experience’, ‘Extremism’, and ‘Mental Health’.

### 3.3 Independent search of key concepts

**Figure 4:**
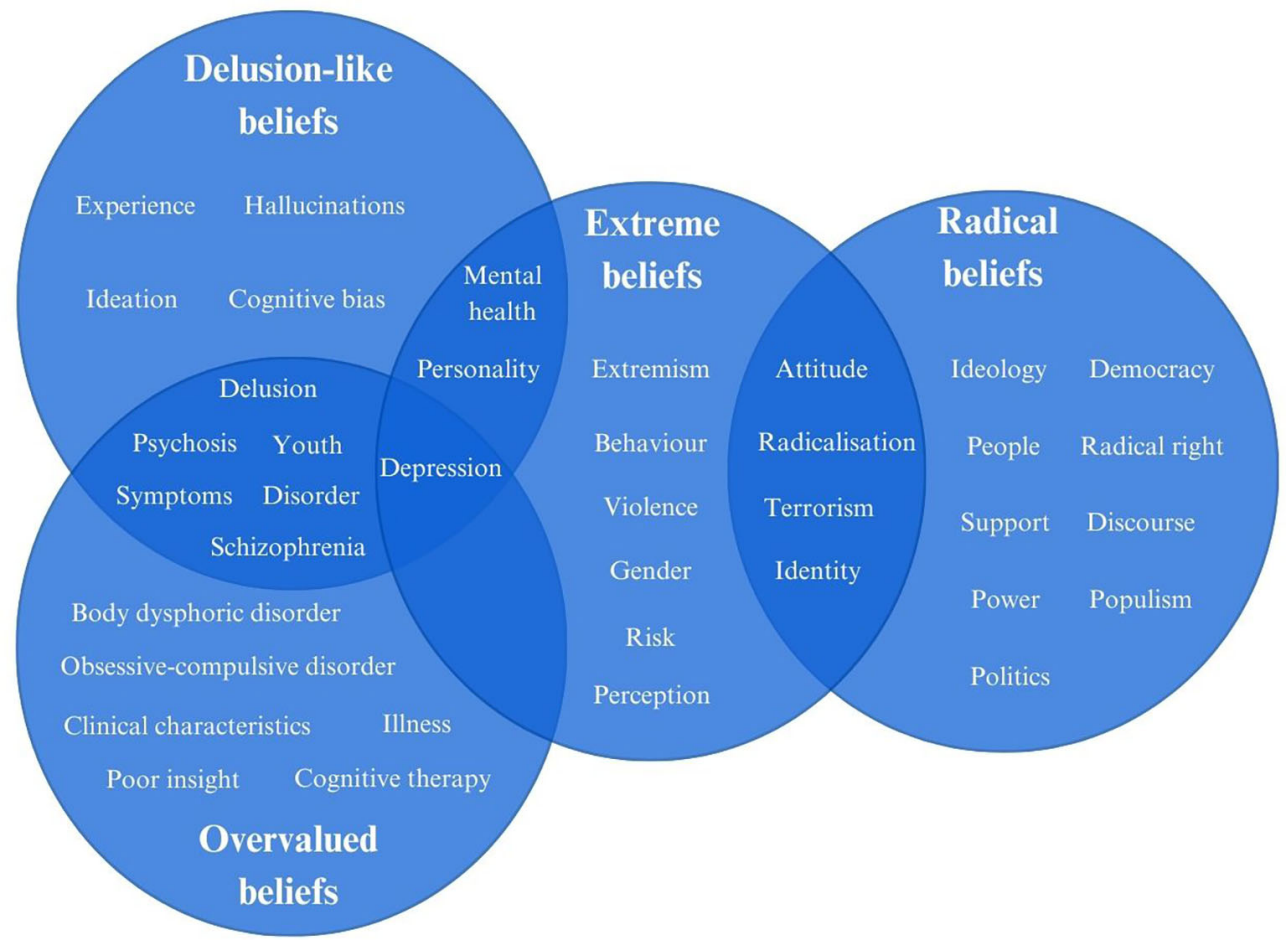
Venn Diagram presenting the top ten strongest keywords for each concept and their overlap across concepts.

*Extreme Overvalued Beliefs and Ideas*. A total of 165 relevant studies were identified. Of those, 97 in psychiatry and medicine, 86 in psychology and neuroscience, and 30 across other social science disciplines. When uploading the results to VOSviewer, 1350 keywords were identified, with 76 meeting the threshold of 4 or more mentions across the studies. **Figure 4** displays a Venn Diagram of the strongest keywords of each concept and how they overlap.

*Delusion-like Beliefs and Strongly Held Ideas*^5^. 160 relevant studies were identified, with 51 in psychiatry and medicine, 71 in psychology and neuroscience, and 73 across social science disciplines. A total of 1,172 keywords were identified, with 39 meeting the threshold of 4 or more mentions across the studies.

*Extreme and Extremist Beliefs*. A total of 2,350 results were identified. Of those, 324 were in psychiatry and medicine, 1,164 in psychology and 1,268 across the social science disciplines. A total of 10,842 keywords were identified, with 785 meeting the threshold of 4 or more mentions.

*Radical Beliefs and Ideas*. A total of 3,073 relevant studies were identified. Of those, 160 were in psychiatry and medicine, 571 in psychology and neuroscience, and 2,537 across other social science disciplines. A total of 11,249 keywords were identified, with 817 meeting the threshold of 4 or more occurrences.

As **Figure 4** presents, there is a noticeable overlap between extreme and overvalued beliefs and delusion-like beliefs, as well as between extreme beliefs and radical beliefs. This is notable, especially considering recent research linking extreme overvalued beliefs and delusion-like beliefs to identity, identification, radicalisation and violent extremism (42, 65, 69, 70).

### 3.4 Social sciences

**Figure 5:**
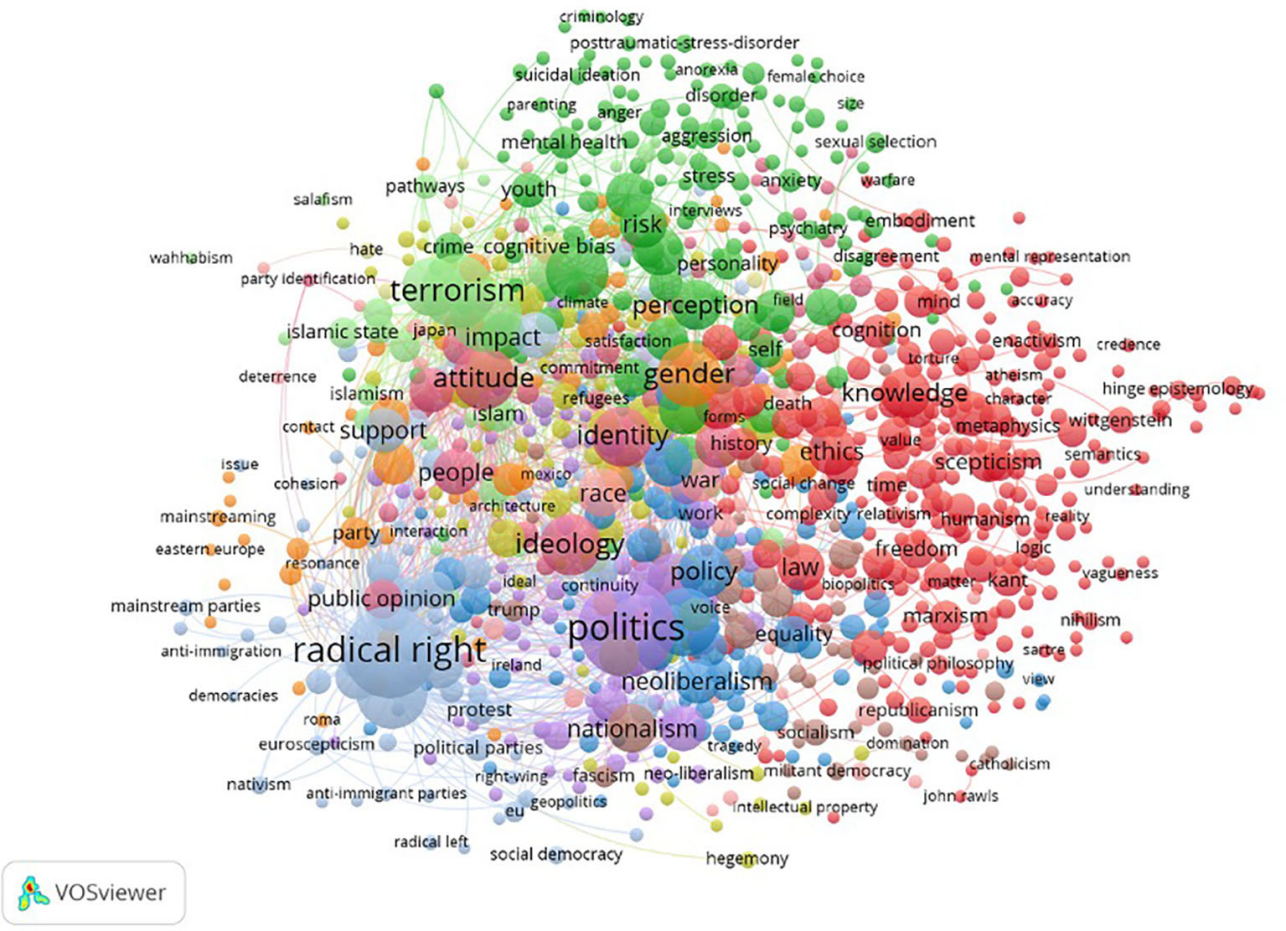
Key-word co-occurrence map for social sciences publications for overvalued, extreme, radical or delusion-like beliefs from 2005 to 2025.

*Note*. There was significant overlap across categories. Mental disorders, medicine, violence and extremism (green), ideologies, politics, and political behaviour (orange, purple, brown and grey); cognition, belief systems, and philosophy (red); identity and attitude (pink), policy and social change (blue).

All concepts were then used for a combined search in law, philosophy, international relations, sociology, criminology, behavioural science, anthropology and other social science disciplines. The total results across these disciplines were 3,397. All results were uploaded to VOSviewer, where a map was created based on bibliographic data, with a focus on keywords across publications. A total of 13,394 keywords were identified, and when a threshold of at least four mentions was implemented, 710 keywords remained. Those were selected and used in the network visualisation presented in **Figure 5**. The top keyword co-occurrences were ‘Politics’, ‘Radical right’, ‘Terrorism’, ‘Radicalisation’, ‘Populism, ‘Democracy’, ‘Gender’, ‘Attitude’. ‘Violence’, ‘Ideology’, ‘Identity’, ‘Extremism’, ‘Policy, and ‘Power’.

### 3.5 Psychiatry, psychology and neuroscience

**Figure 6:**
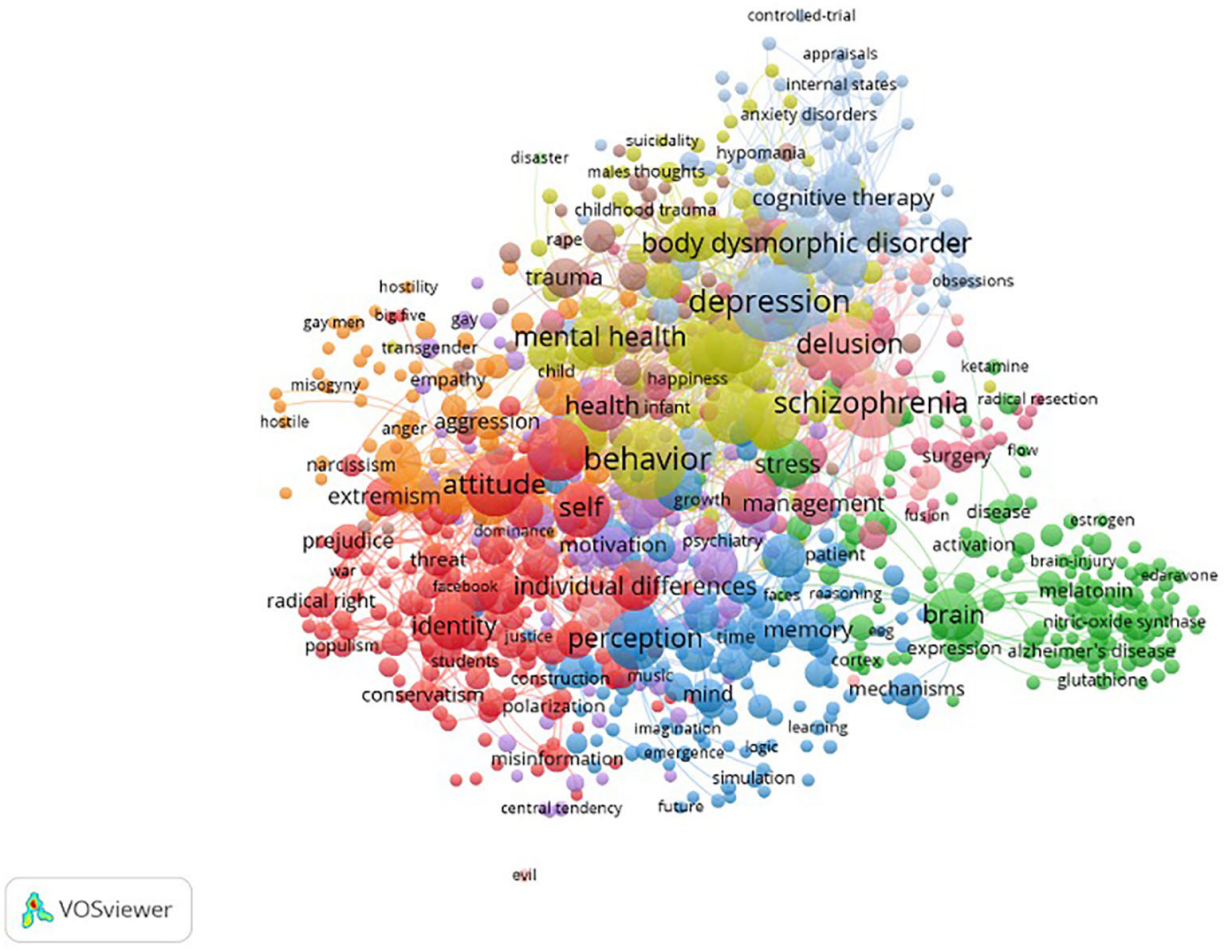
Key-word co-occurrence map in psychiatry and psychology publications for overvalued, extreme, radical or delusion-like beliefs from 2005 to 2025.

*Note*. Mental disorders (grey and pink); identity and ideology (red), cognition and perception (blue); medicine and the human body (green), violence and aggression (orange); overlapping keywords include an intersection of psychiatry and violence (brown) and a category of associated words that overlap significantly across all categories, including behaviour, mental health and happiness (yellow).

The same search was performed in the following disciplines: psychology, psychiatry, neuroscience, legal medicine and medical ethics. A total of 2,117 results were uploaded to VOSviewer. Again, a map based on bibliographic data was generated to explore the co-occurrence of all keywords identified across the research. A total of 10,475 keywords were identified, a threshold of at least four mentions across these publications was set, and a total of 857 keywords remained, and all were used to generate the network visualisation presented in **Figure 6**. The keywords with the strongest link strength in psychiatry and psychology were ‘Depression’, ‘Behaviour’, ‘Schizophrenia’, ‘Attitude’, ‘Personality’, ‘Childhood and Youth’, ‘Perception’, ‘Disorder’, ‘Delusion’, ‘Mental Health’, ‘Risk’, ‘Body Dysphoric Disorders’ ‘Self’, ‘Experience’, ‘Symptoms’, ‘Brain’, ‘Health’, ‘Anxiety’ and ‘Obsessive-Compulsive Disorder’.

The overview of the strongest co-occurring keyword analysis across all disciplines can be partly divided into five overarching focus areas. These categories overlap but remain consistent across research areas. ‘Mental Disorders’ with a focus on delusions, schizophrenia, body dysmorphic disorders and other conditions. ‘Violent Extremism’ with a focus on radicalisation, terrorism and violence. ‘Ideology and Identity’, which included radical right, politics, culture, gender and race. ‘Cognition’ with a focus on perception, emotions, insight and related elements. Lastly, ‘Medicine’ included keywords such as brain, disease and cells. The focus on those categories varied based on terminology and discipline, but the categories overlapped significantly.

## 4 Discussion

This review and bibliometric analysis mapped research on extreme, overvalued, radical and delusion-like beliefs published over the past 20 years. It highlighted related and, at times, synonymous terms and conceptualisations across different scientific disciplines. Due to the interdisciplinary nature of this topic and inconsistencies in terminology, conducting a bibliometric review sheds light on current and past developments and highlights limitations, gaps and possible opportunities for future research. There was a gradual increase in publication rates from 2005 to 2025, with the exception of 2015, and then remaining relatively stable from 2020, with most papers being published in the past 10 years. The majority of publications were from the United States and England, which accounted for around half of all publications, however, this is likely due to the focus on English research.

As presented in the findings, there were discrepancies in the co-occurring keywords across the concepts and disciplines. Extreme and overvalued beliefs, delusion-like beliefs and strongly held ideas had great overlaps, mostly concerning mental illness diagnoses, such as delusions, psychosis and schizophrenia. Extreme, extremist and radical beliefs had a clear focus related to radicalisation, identity and terrorism. While psychiatry and psychology focused predominantly on medical factors and mental disorders, other social science disciplines focused mainly on terrorism, radicalisation and violent extremism. However, as noted, this changed slightly in the past ten years, where the focus in psychology and psychiatry shifted towards trends observed in other social science disciplines, with terrorism, radicalisation and extremism rising significantly. Surprisingly, little focus was observed on personality abnormalities, despite being described as a vital factor in extreme overvalued beliefs (22). Similarly, neurodivergence was not strongly associated with any of the terms, despite repeated mention in coverage of lone-actor attacks, violent extremism and fixation (95, 96).

There appeared to be an increased focus in psychology and psychiatry on identity, personality, extremism and violence in recent years, suggesting a growing interest in exploring overvalued, extreme and delusion-like beliefs in medicine outside of traditional diagnostic categories. This is in line with current emerging themes where fixation in threat assessment and treatment of fixated offenders has been a growing focus in both forensic psychiatry and psychology (17, 40). Not only are these beliefs important in the context of violence, but they are also relevant when exploring the deterioration in the social and occupational functioning of young people, associated with isolation, participation in hate speech and bigotry, and adherence to violent ideologies, conspiracy theories and extreme political views (20–22, 65, 72, 97–100).

This has been observed in young men who identify as “Incels”, a misogynistic online community that played a crucial role in the Isla Vista shooting and the Toronto van attack, as well as multiple attacks by self-proclaimed sovereign citizens who have attacked law enforcement, healthcare workers and government personnel (22, 50, 64, 65, 99, 101). These cases illustrated how extreme, overvalued or delusion-like beliefs can spread in online ecosystems and develop into a fixation (34). While different scientific disciplines have used different terminology and concentrated on different aspects of extreme, overvalued and delusion-like beliefs, some of the underlying characteristics remain similar. Future research should encourage cross-disciplinary dialogue and build on the multiple perspectives across psychiatry, psychology and social science disciplines. This might lead to valuable new observations and findings, given that many of the concepts overlap significantly and their application might not be as narrow as often described, such as overvalued ideas in Anorexia Nervosa (64). Broadening the application and applying the knowledge in different circumstances can improve our understanding, not only of violent extremism and lone-actor grievance-fuelled violence, but in multiple scenarios where extreme, overvalued and delusion-like beliefs shape behaviour and decision-making.

Importantly, reaching a common language of overvalued beliefs and fixation could have the potential to partially bridge the gap currently emerging in threat assessment tools and studies on violent extremism, where offenders have no clear ideology that drives their fixation. This paper has sought to lay a foundation for cross-disciplinary threat assessment research by highlighting conceptual and definitional discrepancies and overlaps across disciplines. The incoherent combination of beliefs shared with subcommunities online is a reappearing risk factor in lone-actor grievance-fuelled violence, where both fixation and grievances have been highlighted (19, 38). The conceptualisation of extreme overvalued beliefs helped draw on decades of research in psychiatry and psychology, providing a common framework for non-delusional fixation shared with a subcommunity at risk of inspiring targeted violent attacks (70). The concept has clear commonalities with overvalued, delusion-like, extreme, and radical beliefs, which have long been a recognised risk factor for lone-actor violence (17, 36) and highlighted as a proximal warning behaviour for lone-actor terrorism (44, 102).

### 4.1 Limitations, Debate and Future Direction

While this paper was a first attempt to capture the cross-disciplinary commonalities and differences in research on extreme, overvalued and delusion-like beliefs, some limitations should be noted. The first challenge stems from the clear lack of consensus on what is being explored across studies and disciplines, which needs to be addressed in future research. Overvalued and delusion-like beliefs were more commonly mentioned in psychiatry and psychology, with a strong focus on medical diagnosis and implications. While overvalued and delusion-like beliefs have also been explored in the context of violent extremism and lone-actor grievance-fuelled violence (21, 50, 65, 72, 103), it appears that extreme and radical beliefs were more common in that context. This points to the significant discrepancies in terminology, which we highlighted earlier, and requires further exploration (50, 54, 55, 58, 73). As previously mentioned, the concepts are, at times, also used interchangeably and, occasionally, have partially distinct meanings with less specific terminology. Agreeing on consensus definitions would be a crucial step towards improving foundational research on extreme, overvalued and delusion-like beliefs.

Similarly, other concepts could potentially have been included in the current review, including ‘radicalisation’, ‘ideologies’ or certain subgroups within the ideology, such as the aforementioned ‘Incels’, ‘QAnon’, or ‘Sovereign Citizens’. Due to a lack of consensus on terminology, some researchers may opt for a clear connection to the content of the belief, which could, at times, be synonymous with delusion-like, radical, extreme or overvalued beliefs. However, as the aim of this review was to explore existing knowledge on these beliefs, we only explored the overarching concepts and terminologies that have a clear overlap. Furthermore, the search was only performed using Web of Science, following the methodology of similar studies (87–90). It is possible that some publications, not captured in Web of Science, were not included in the review and analysis. To minimise the effects of this limitation, a partial cross-database validation was conducted. However, the results from the secondary database were not included in the analysis, meaning that the results relied on a single database to highlight and analyse existing literature. While this is not optimal, it was determined to be appropriate given the discipline-specific focus of the review, allowing for a more detailed and specific analysis of the literature. While acknowledging this limitation, the breadth and depth of studies from diverse disciplines available through Web of Science (88, 89) would be sufficient to offer a representative sample of studies for the purpose of our research questions. Nevertheless, including multiple databases in future research would be valuable, as results from a single database can be biased. This could be particularly important for reviews focusing on all publications across scientific disciplines, with an emphasis on academic institutions, contributing authors and time periods. Lastly, we recognise a similar limitation in restricting our review to publications in English, which skewed the geographical distribution of the publications. While certain publications may have been missed when applying this criterion, the bibliometric analysis did present a comprehensive overview of the topic across disciplines and distinct concepts.

Although our results shed light on the current patterns and gaps for future research, the topic remains difficult to navigate due to its inherent complexities. Radical and extreme beliefs can take on separate meanings depending on several contextual factors (96, 104). Labelling a belief as radical or extreme might not be generalisable to other cultures, periods or situations (104). However, while both content and perceptions of these ideologies can differ across time and space, the psychology of extreme ideologies remains similar (105). Our findings imply that forensic experts across disciplines should start applying a shared vocabulary to further our understanding of the aetiology, prevalence, urgency, and possible co-occurring disorders of extreme, overvalued and delusion-like beliefs. This will also allow researchers to explore these concepts within a variety of violent subcultures and ideologies (106) and develop comprehensive and interdisciplinary frameworks to address complex clinical and legal issues.

While the risks stemming from overvalued beliefs have been recognised (36, 50, 65, 66), they present novel challenges to forensic evaluations and clinical treatment, especially in light of modern technological advances (72, 73). As the research focus has been divided across disciplines, and given current developments in this field, some perspectives warrant more attention. Fixated offenders have higher rates of mental illness, but that does not necessarily equal irrational thinking, nor does it fall under the M’Naghten rules for legal insanity (107). Therefore, future research must take the role of mental illness into account, as has been done in psychiatry and psychiatry, while still focusing on other contextual factors. Some notable factors highlighted in this review include cognitive biases, personality abnormalities, the role of grievances and changes in identity, emotions and perception (22, 34, 38, 65, 72, 73). It is evident that emerging threats are changing, but research has consistently found that attributing radicalisation pathways towards violence solely to mental illness (96, 108), specific ideologies or radicalisation (109) remains insufficient. Attempts to present a profile or a singular pathway leading an individual to violent extremism have fallen short (110). However, this is not only notable in the individual pathways that escalate towards extreme violence but also due to various other adverse outcomes for the individual and society that are of concern for researchers, psychiatrists and psychologists and require more nuanced assessments and protocols (65, 72, 73). Lastly, while acknowledging the importance of monitoring hateful or violent beliefs and ideologies, the content itself might not be the only relevant indicator of threat, but the extent to which the individual fixates on it. Thus, future research across disciplines should not solely focus on *what* the individual believes, but *how* it is believed.

## Data Availability

The original contributions presented in the study are included in the article/**Supplementary Material**. Further inquiries can be directed to the corresponding author/s.

## Footnotes

1 While the cross-database comparison was largely consisted, there were some observed differences which underscore the importance of considering metric variations across databases moving forward. The total results across the applied exclusion/inclusion criteria in review remained above 70% for all metrics, except for top contributing affiliations (67%) and highest cited articles (50%). See a more detailed coverage and exploration of the partial cross-database validation in the **Supplementary Material**.

2 While acknowledging that these terms are not entirely synonymous (96), for the sake of a bibliographic review, they were mapped using the same cluster.

3 Note that this analysis only contains early September of 2025, and if the trend continues, the number of publications is expected to rise from 2024.

4 It is important to note that this representation is skewed as this review excluded non-English publications. This limitation further highlighted in the discussion.

5 The DSM-5 presents strongly held ideas as distinct non-delusional phenomena in the chapter on delusions (p. 85).
