# Supplementary material for: Pathological fixation on shared beliefs: a review and bibliometric analysis of extreme, overvalued and delusion-like beliefs
Source: Front Psychiatry. 2025 Dec 4;16:1715886. doi: 10.3389/fpsyt.2025.1715886 (PMC12711862; doi:10.3389/fpsyt.2025.1715886)
Supplement: Supplementary file 1 [file Supplementaryfile1.docx]

Partial cross-database validation for *Pathological Fixation on Shared Beliefs: A Review and Bibliometric Analysis of Extreme, Overvalued and Delusion-like Beliefs*

|  | **Web of Science Core Collection (primary)** | **SCOPUS (secondary)** | **Cross-source comparison** |
| --- | --- | --- | --- |
| **Total results** | 45,451 | 63,950 | 71% |
| **English only** | 41,196 | 58,252 | 71% |
| **2005-2026** | 37,961 | 51,303 | 74% |
| **Publication year** | 2025 (2,109)  2024 (3,221)  2023 (3,125)  2022 (3,102)  2021 (2,792)  2020 (2,519)  2019 (2,314)  2018 (2,219)  2017 (1,988)  2016 (1,950)  2015 (1,847) | 2025 (2,982)  2024 (4,357)  2023 (4,204)  2022 (3,834)  2021 (3,538)  2020 (3,180)  2019 (3,004)  2018 (2,732)  2017 (2,529)  2016 (2,419)  2015 (2,202) | Same distribution |
| **Top countries** | 1. **United States (12,473)** 2. **China (7,206)** 3. **United Kingdom (4,147)** 4. **Germany (2,921)** 5. **Canada (2,023)** 6. **Italy (1,986)** 7. **Australia (1,846)** 8. **France (1,781)** 9. **India (1,646)** 10. **Russia (1,584)** 11. **Spain (1,497)** 12. **Japan (1,374)** 13. **Netherlands (1,104)** 14. **Sweden (787)** 15. South Korea (784) | 1. **United States (17,181)** 2. **China (8,384)** 3. **United Kingdom (7,047)** 4. **Germany (3,422)** 5. **India (2,537)** 6. **Canada (2,524)** 7. **Italy (2,441)** 8. **Australia (2,357)** 9. **France (2,126)** 10. **Russia (1,806)** 11. **Japan (1,710)** 12. **Spain (1,646)** 13. **Netherlands (1,308)** 14. Brazil (906) 15. **Sweden (357)** | The same top 15 countries, with the exception of Brazil (SCOPUS) and South Korea (WoS). Both were ranked 16th on the other database. |
| **Top affiliations** | 1. **University of California (1,097)** 2. **Chinese academy of sciences (960)** 3. **CNRS (814)** 4. **University of London (743)** 5. **Russian Academy of Sciences (519)** 6. University of Texas (449) 7. Harvard University (441) 8. University of Ohio (414) 9. University of Florida (354) 10. **University of Oxford (343)** 11. **University College London (288)** 12. ETH Zurich (275) 13. **University of Cambridge (274)** 14. **University of Toronto (253)** 15. **University of Michigan (248)** | 1. **Chinese Academy of Sciences (860)** 2. **CNRS (613)** 3. **University of Oxford (416)** 4. **Russian Academy of Sciences (399)** 5. **University of Cambridge (359)** 6. **University of California, Berkeley (Berkeley: 344; Los Angeles: 245)** 7. University of Chinese Academy of Arts (335) 8. **University College London (304)** 9. **University of Toronto (288)** 10. **University of Michigan (273)** 11. **King’s College London (247)** 12. Stanford University (246) 13. Columbia University (233) 14. University of Melbourne (233) 15. University of Manchester (228) | 67% overlap |
| **Top researchers** | 1. **Alberto Briganti (37)** 2. **Pierre I. Karakiewicz (37)** 3. **Francesco Montorsi (31)** 4. **Shahrokh F. Shariat (31)** 5. **Russel J Reiter (30)** 6. **Michael J Davies (26)** 7. **AK Srivastava (37)** 8. **Markus Graefen (23)** 9. **Yang Lee (21)** 10. Mani Menon (19) | 1. **AK Srivastava (47)** 2. **Pierre I. Karakiewicz (40)** 3. **Alberto Briganti (35)** 4. **Russel J Reiter (30)** 5. **Shahrokh F. Shariat (30)** 6. **Michael J Davies (29)** 7. **Francesco Montorsi (27)** 8. **Markus Graefen (20)** 9. **Yang Lee (20)** 10. N.J. Groenewald (19) | 90% overlap  Same top contributing authors, with the exception of Menon (WoS) and Groenewald (SCOPUS). |
| **Top journals** | 1. **Chemical Engineering Journal (206)** 2. **Proceedings of SPIE (204)** 3. **Plos One (177)** 4. **Astronomy and Astrophysics (163)** 5. **Astrophysical Journal (160)** 6. **Scientific Reports (156)** 7. **Journal of the American Chemical Society (133)** 8. **Communications in Algebra (114)** 9. **Lecture Notes in Computer Science (113)** 10. **Monthly Notices of the Royal Astronomical Society (113)** 11. Macromolecules (109) 12. **ACS Applied Materials and Interfaces (107)** 13. **Separation and Purification Technology (97)** 14. **Physical Chemistry Chemical Physics (96)** 15. **Religions (86)** | 1. **Proceedings of SPIE (304)** 2. **Chemical Engineering Journal (204)** 3. **Lecture Notes in Computer Science (179)** 4. **Plos One (167)** 5. **Astronomy and Astrophysics (152)** 6. **Scientific Reports (147)** 7. **Astrophysical Journal (131)** 8. **Journal of the American Chemical Society (129)** 9. **ACS Applied Materials and Interfaces (108)** 10. International Journal of Biological Macromolecules (100) 11. **Communications in Algebra (98)** 12. **Separation and Purification Technology (93)** 13. **Monthly Notices of the Royal Astronomical Society (90)** 14. **Physical Chemistry Chemical Physics (84)** 15. **Religions (82)** | 93% overlap  The same top journals, with the exception of Macromolecules (WoS) and International Journal of Biological Macromolecules (SCOPUS). |
| **Most cited articles** | 1. **The role of deliberate practice in the acquisition of expert performance, Anders Ericsson et al., (1993)** 2. Inhibition of anaerobic digestion process: A review, Chen et al. (2008) 3. **Naming materials in the magma/igneous rock system, Middlemost (1994)** 4. **The glutathione s-transferase supergene family: Regulation of GST and the contribution of the lsoenzymes to cancer chemoprotection and drug resistance Part I, Hayes & Pulford (1995)** 5. **Bilirubin is an antioxidant of possible physiological importance, Stocker et al. (1987)** 6. Topological photonics, Ozawa et al. (2019) 7. **ReaxFF reactive force field for molecular dynamics simulations of hydrocarbon oxidation, Chenoweth et al., (2008)** 8. A network Theory of Mental Disorders, Borsboom (2017) 9. Dehumanization: An integrative review, Haslam (2006) 10. Flexible high-temperature dielectric materials from polymer nanocomposites, Li et al. (2015) | 1. Extreme learning machine: Theory and applications, Huang et al. (2006). 2. **The role of deliberate practice in the acquisition of expert performance, Anders Ericsson et al., (1993)** 3. Steric Effects of Phosphorus Ligands in Organometallic Chemistry and Homogeneous Catalysis, Tolman (1977) 4. **Naming materials in the magma/igneous rock system, Middlemost (1994)** 5. **The glutathione s-transferase supergene family: Regulation of GST and the contribution of the lsoenzymes to cancer chemoprotection and drug resistance Part I, Hayes & Pulford (1995)** 6. DNA sequence analysis with a modified bacteriophage T7 DNA polymerase, Tabor & Richardson (1987) 7. **Bilirubin is an antioxidant of possible physiological importance, Stocker (1987)** 8. Toward a field of intersectionality studies: Theory, applications, and praxis, Cho et al. (2013) 9. **ReaxFF reactive force field for molecular dynamics simulations of hydrocarbon oxidation, Chenoweth et al., (2008)** 10. Analysis of the geometry of the hydroxymethyl radical by the different hybrids for different spins natural bond orbital procedure, Carpenter & Weinhold (1988) | 50% overlap |
| **Results after applying all criteria (document type, date and language but excl. subject areas)** | 35,085 | 46,327 | 76%   The discipline-specific criteria were not applied. It would not be an accurate representation as the categories on SCOPUS are broader than on WoS. |

- While the comparison rate was above 50% in all categories, SCOPUS displayed a higher volume of results than Web of Science Core Collection. Web of Science has a focused approach on top-tier journals, whereas SCOPUS has a larger database (Hoang, 2025), with SCOPUS having 44,534 active journals (*Scopus Sources*, n.d.), while Web of Science Core Collection has 22,774 active journals (*Web of Science Master Journal List,* n.d.). In addition, while SCOPUS displayed a higher number of results, the distribution by year and contributing countries was similar.
- The top contributing institutions and most cited articles had a 53-67% similarity. The discrepancies are likely due to the higher number of results displayed on SCOPUS.
- A partial cross-database validation was conducted due to the discipline-specific nature of our analysis, whereas Web of Science allows a more specific filtering. However, including a secondary database is the ideal approach for bibliometric reviews. We recognise and highlight this in our discussion, but also emphasise that Web of Science is frequently utilised in bibliometric reviews and was determined to be sufficient to meet the research objectives of exploring keyword co-occurrences across scientific disciplines.
- Despite some limitations of a partial cross-database validation, Web of Science was deemed more suitable for the bibliometric review because of its filtering options. Our review aimed to explore focus areas across scientific disciplines and to accurately examine the co-occurrence of keywords, which required a clear specification of the research field. This was not achievable with SCOPUS, which provides broader categories, such as ‘Medicine’ instead of specific fields like ‘Psychiatry’, ‘Medical ethics’, and ‘Legal Medicine’. Although the findings from SCOPUS were tested using VOSviewer, the lack of specificity in filtering resulted in overly broad results that could not meaningfully contribute to the existing literature on extreme, overvalued, and delusion-like beliefs. Furthermore, due to its focus on top-tier journals and scientific publications, Web of Science does include the most prominent journals in the Social Sciences, Medicine, and Psychology, which were the focus of our review. While merging databases can offer significant benefits for bibliometric reviews (Caputo & Kargina, 2022; Hoang, 2025), the specific aims of our review made a focused analysis of a single database the most appropriate approach. Nonetheless, we recognise this limitation and acknowledge that searching other databases could be advantageous in future research, particularly for analysing all publications with an emphasis on academic institutions, contributing authors and time periods.
- To mitigate any other potential limitations, we followed appropriate guidelines to ensure the quality of the analysis for the bibliometric review, while taking the methodology and objectives of our review into account. Specifically, we set clear research objectives when determining our methodology, described the selection process clearly, including the identification of papers (keywords, Boolean strings, language used), screening (filtering, languages, eliminating certain documents) and eligibility (what was eliminated and recognising the scope and breadth of Web of Science as a primary database) (Hoang, 2025). Our results have also been updated to include literature published through mid-September 2025 to ensure the most up-to-date representation of current research.

References

Caputo, A., & Kargina, M. (2022). A user-friendly method to merge Scopus and Web of Science data during bibliometric analysis. *Journal of Marketing Analytics*, *10*(1), 82–88. https://doi.org/10.1057/s41270-021-00142-7

Hoang, A.-D. (2025). Evaluating Bibliometrics Reviews: A Practical Guide for Peer Review and Critical Reading. *Evaluation Review*, 0193841X251336839. https://doi.org/10.1177/0193841X251336839

*Scopus preview—Scopus—Sources*. (n.d.). Retrieved 13 September 2025, from https://www.scopus.com/sources.uri

*Web of Science Master Journal List—Search*. (n.d.). Retrieved 13 September 2025, from https://mjl.clarivate.com/search-results
